# Investigating functional consistency of mobility-related urban zones via motion-driven embedding vectors and local POI-type distributions

**DOI:** 10.1007/s43762-022-00049-8

**Published:** 2022-06-28

**Authors:** Alessandro Crivellari, Bernd Resch

**Affiliations:** 1 Department of Computer Science and Engineering, Southern University of Science and Technology, Shenzhen, 518055 China; 2 Department of Geoinformatics, University of Salzburg, 5020 Salzburg, Austria; 3 Center for Geographic Analysis, Harvard University, Cambridge, MA 02138 USA

**Keywords:** Human mobility, Points of interest, Functional regions, Urban areas, Embeddings, Neural networks, Unsupervised learning

## Abstract

Urban morphology and human mobility are two sides of the complex mixture of elements that implicitly define urban functionality. By leveraging the emerging availability of crowdsourced data, we aim for novel insights on how they relate to each other, which remains a substantial scientific challenge. Specifically, our study focuses on extracting spatial-temporal information from taxi trips in an attempt on grouping urban space based on human mobility, and subsequently assess its potential relationship with urban functional characteristics in terms of local points-of-interest (POI) distribution. Proposing a vector representation of urban areas, constructed via unsupervised machine learning on trip data’s temporal and geographic factors, the underlying idea is to define areas as “related” if they often act as destinations of similar departing regions at similar points in time, regardless of any other explicit information. Hidden relations are mapped within the generated vector space, whereby areas are represented as points and stronger/weaker relatedness is conveyed through relative distances. The mobility-related outcome is then compared with the POI-type distribution across the urban environment, to assess the functional consistency of mobility-based clusters of urban areas. Results indicate a meaningful relationship between spatial-temporal motion patterns and urban distributions of a diverse selection of POI-type categorizations, paving the way to ideally identify homogenous urban functional zones only based on the movement of people. Our data-driven approach is intended to complement traditional urban development studies on providing a novel perspective to urban activity modeling, standing out as a reference for mining information out of mobility and POI data types in the context of urban management and planning.

## Introduction

Urban morphology and human mobility have been examined extensively within the context of urban planning and management. Whereas often treated as separate entities, the study of their potential relationship remains a substantial scientific challenge, involving analyses of complex interactions between built environment and urban processes such as navigation, daily routine activities and long-term life choices (Earnhart, 2001; Handy et al., 2002; Hillier & Iida, 2005). A particular case refers to the definition and investigation of urban functional zones, namely city areas characterized by a certain internal homogeneity in their complex mixture of local activities, primarily derived from human mobility processes and social behaviors (Crooks et al., 2015; Stead & Marshall, 2001; Zhang, 2004). Recent works highlighted their importance for planning applications, transportation design, and further social studies (Gao et al., 2013; Liu, Gong, et al., 2015; Yuan et al., 2014).

The challenge of our work consists of capturing high-level latent functional concepts by solely analyzing human motion behaviors, instead of urban physical characteristics. Towards this goal, we leverage the emerging availability of crowdsourced data, making use of geolocated information to gain novel insights into functional aspects of urban spaces. The underlying city structure, indeed, goes beyond the only physical environments, presenting internal interactions among urban areas, which lead to depict dynamic systems rather than static states. Urban functionalities are not only related to separate physical entities, but revolves around an integrated flow of spatial influences between locations (Berry, 1968). Most studies focusing on urban morphologies do not directly capture human dynamics within city areas, missing a prominent insight on the development of urban functionalities (Liu, Gong, et al., 2015). The traditional objective geographic separation fails to capture perceptual boundaries and inherent actual “usage” of the urban territories, which may differ according to the complex human dynamics and motion activities (Zhou & Zhang, 2016). This research provides a bottom-up view on the exploration of complex intra-city mobility flows and their relation with urban functional characteristics.

The proliferation of location-aware devices and technologies has boosted the collection of data with a spatial content, often publicly shared and widely disseminated (Batty et al., 2010; Batty et al., 2012). These data represent the basis for bottom-up approaches that gather first-hand information rather than assuming trends from theoretical assumptions, further allowing for combinations of different sources that contribute to describe cities as integrated systems of multiple interacting networks. In this context, mobility data are valuable factors for modeling urban structures and human activities. Previous studies involved the use of taxi data (Liu, Gong, et al., 2015), mobile phone traces (Pei et al., 2014; Toole et al., 2012), and social-media check-ins (Zhou & Zhang, 2016). In line with this expanding trend, our research aims to contribute towards the spatial-temporal understanding of urban mobility and the investigation of possible correlations between travel patterns and urban functions (Liu, Gong, et al., 2015).

Specifically, we leverage taxi trip data as a raw source of information on the urban distribution of points-of-interest (POI) typologies. Although the purpose of taxi trips can be very diverse, it is largely affected by the interaction with urban internal functions (Dong et al., 2019; Zhao et al., 2020); therefore, hypothetically, urban mobility can be used to identify homogeneous regions with similar functional characteristics. Our paper follows this direction by using urban POI data and taxi travel demand to explore the relationship between human motion and the mixture of basic functional units across the urban space (Gao et al., 2019; Wang et al., 2019). The research goal is to perform an automatic mining of spatial-temporal characteristics from nearly-raw taxi data, without requiring any manual feature extraction or human knowledge assistance, and subsequently investigate the outcomes with respect to the POI-type distribution throughout the urban environment.

We hereby propose a novel framework based on similarities between multi-dimensional vector representations of urban areas, constructed via unsupervised machine learning on trip data’s temporal and geographic factors. The underlying idea is to define urban areas as “related” if they often act as destinations of similar departing regions at similar points in time, regardless of any other explicit information. The effective implementation leverages the concept of embeddings, real-valued dense vectors originally introduced in the natural language processing (NLP) domain for modeling semantic relations between words (Bengio et al., 2000; Mikolov, Chen, et al., 2013; Mikolov, Sutskever, et al., 2013; Pennington et al., 2014) and subsequently adopted, in very recent years, also for geographic and urban applications (Liu et al., 2019; Qiu et al., 2019; Yao et al., 2017; Zhai et al., 2019). Main adaptations in literature comprise embedding representations of locations or POIs originating from their relative spatial distribution over the territory (Liu et al., 2020; Yan et al., 2017) or derived from the way human trajectories cross them (Crivellari & Beinat, 2019; Zhou et al., 2018). Our specific case aims to model hidden relations within the generated vector space in a completely data-driven manner, whereby areas are represented as points and stronger/weaker semantic relatedness is conveyed through their relative distances. These output vectors are intended to implicitly map the mobility relatedness between geographic regions, potentially identifying homogenous functional areas across the city.

The algorithm relies on two processing steps: organizing trip destination areas into spatial-temporal sequences, and employing a Word2vec-based model to accordingly generate their embedding representations. This mobility-related outcome is then compared with the POI-type distribution across the urban environment, to assess the functional consistency of mobility-based clusters of urban areas. In fact, independently from the motion embeddings, a further vector representation of urban areas is parallelly defined, modeled in the form of POI distributions, as urban POIs reflect, to some extent, the spatial allocation of urban functions and travel purposes (Bao et al., 2020; Miaoyi et al., 2018).

Evaluated on a recent open-sourced dataset of taxi travel demand in New York City, results indicate a meaningful relationship between spatial-temporal motion patterns and urban distributions of a diverse selection of POI-type categorizations, paving the way to ideally identify homogenous urban functional zones only based on the movement of people. The meaningful insights of our data-driven approach are intended to complement traditional urban development studies on providing a novel perspective to urban activity modeling, standing out as a reference for automatically mining information out of different data types in the context of urban management and planning.

## Methodology

We designed an unsupervised approach for constructing mobility-based multi-dimensional dense vectors (embeddings) of urban areas. These output vectors are intended to implicitly map the mobility relatedness between geographic regions, aiming to identify homogenous functional areas across the city. The algorithm relies on organizing trip destination areas into spatial-temporal sequences, and employing a Word2vec-based model to accordingly generate embedding representations of the elements along such sequences. Concurrently, further POI-based vectors are separately defined, reflecting the urban POI-type distribution over the territory.

The global workflow can be summarized in two parallel processing steps (P1, P2), subsequently converging into two evaluation steps (E1, E2):

- P1. Trip data processing: from raw taxi trip data to sequences of urban areas (Section 2.1.), to Word2vec sequential modeling for generating mobility embeddings (Section 2.2.);
- P2. POI data processing: POI spatial distribution and categorization modeling for generating POI-type vectors of urban areas (Section 2.3.);
- E1 (on P1). Mobility-based evaluation: motion-related investigation based on similarity metrics among the generated mobility embeddings of urban areas (Section 3.3.1.);
- E2 (on P1+P2). Functional evaluation: functional consistency within mobility-related urban areas based on similarity metrics of POI-type vectors according to mobility-based regional clusters (Section 3.3.2.).

### Trip data pre-processing

Trip recordings are represented as space-time events, defined by the geographic origin and destination areas, and the corresponding time stamp: *T*_*i*_ = (*Origin*_*i*_, *Destination*_*i*_, *t*_*i*_). Depending on the data source, additional information may be present; however, aiming for an extensive application, we solely rely on the above-mentioned attributes.

The pre-processing phase is directed to transforming single origin-destination trips into a collective sequential data format. The idea is to define ordered sequences of urban areas over time, connecting destination regions based on similar space-time characteristics of their starting points. These sequences of urban areas then make up the training corpus of the following Word2vec model.

The sequence definition is based on a distinctive procedure. Given an urban territory divided into a set of urban areas, each trip-related origin area is used to define a separate characteristic sequence. The sequence contains chronologically ordered destination areas associated to that same origin area, whereby each element in the series is reported in the form of a pairing (*Area* _ *ID*_*i*_, *t*_*i*_). A certain area *j*, therefore, comprises a sequence *S*_*j*_ = {(*Area* _ *ID*_*i*_, *t*_*i*_) | *i* = 1, 2, 3, …}_*j*_: time information is explicitly encoded in the sequence, together with the unique identifier of the destination areas. The collection of such sequences represents the effective input data format for the vector-based representation learning process. In the next subsection, we introduce the Word2vec implementation and present its adapted version to the proposed mobility-based embedding generation task.

### Embedding model for generating vector representations of urban areas

#### Word2vec algorithm

The rise of embedding vector representations is originally ascribed to the NLP domain, whose use pertains the inherent definition of semantic relations between words, based on the direct sequential processing of raw text. This concept has been gradually adapted to a variety of research domains related to sequential data analysis, generalizing the definition of “words” into any format of “categorical entities”, and the definition of “sentences” into any sequential organization of such entities implicitly describing their dependencies.

In broad terms, embeddings are identified as dense vectors of “meaning”, whose representation is derived from the distribution of entity co-occurrences in a large training corpus. The underlying intuition presumes that entities occurring in similar contexts share similar vector representations.

The most used embedding generation procedure is the Word2vec model (Mikolov, Sutskever, et al., 2013), an artificial neural network structure made of a single linear projection layer between the input and the output layers. The model is explicitly designed for learning entity representations from sequential data, and is therefore generally considered an unsupervised approach. However, in practice, its training process is guided by an internal auxiliary prediction task. Given a set of pre-defined distinctive entities, organized into a training corpus made of a collection of sequences, the algorithm is intended to scan each sequence with a sliding window and, at each step, attempting on predicting the current entity with the help of its neighbor entities along the sequence (or vice versa, depending on which of the two original versions is implemented). The prediction outcomes during the training process are aimed to provide an according update of the network weights. In particular, the embedding vectors are specifically represented by those weights connecting each entity in the input layer to the neurons of the hidden layer (the totality of embedding vectors therefore refer to a weight matrix of dimensionality *num_entities × hidden_size*). Prediction here is therefore not an aim in itself, but a proxy to learn vector representations.

Our implementation resorts to the Skip-gram approach, defining a learning process focused on maximizing, at each sliding step, the probability of predicting the neighbor entities *nE*_1_, …, *nE*_*j*_, along the sequence, of a given focus entity *E*_*t*_, with regard to its embedding θ_*t*_. The cost function *C* represents the negative log probability of the correct prediction, optimized with mini-batch stochastic training:

$$\mathrm{C}=-{\sum}_{i=1}^j\log p({nE}_i\left|{E}_t\right)$$

The resulting gradient, derived with respect to the embedding parameters θ (i.e., ∂ *C* /∂ θ), triggers the update of their values. By repeating the process over the entire training corpus until convergence, the corresponding vectors of all entities are learned, giving rise to a multi-dimensional vector space inherently mapping semantic relations through the relative distance between entities.

#### Model training and vector generation

The pre-processed data, in the form of origin-specific sequences of chronologically ordered destination areas, serve as a training corpus, whereby each element in the sequence is defined as a pairing (*Area* _ *ID*_*i*_, *t*_*i*_). The totality of unique urban areas included in the corpus identifies the “vocabulary” set, which is intended to be translated into corresponding embedding vectors. A characteristic vector representation is therefore associated to each of the areas, together composing an embedding matrix of size *num_areas × vector_size*.

**Fig. 1:**
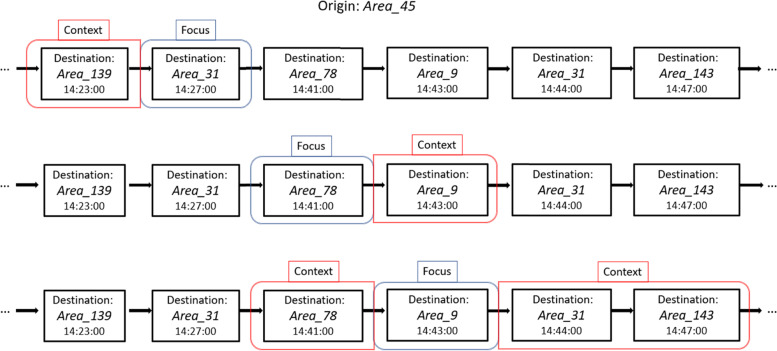
Word2vec sliding window training process with a window size of five minutes in the past and five in the future

The matrix is updated following the Skip-gram training process. Each sequence is scanned with a sliding window, progressively targeting, at each step, a focus area and its context, input and output variables to the Word2vec model, respectively. The mobility relatedness between urban areas is therefore translated, in practice, into consistent vector representations built on time-dependent destination co-occurrences along space-dependent sequences of origin areas. The context of each destination area is defined based on the temporal proximity of other destinations along the same sequence, which characterizes a certain origin area. This means relating destination areas of trips occurring at a similar point in time, and originating from the same initial area. The temporal proximity is captured through a time-dependent sliding window, defining a variable-length context. In contrast to the traditional Word2vec model, setting the window hyperparameter as a pre-defined fixed number of context elements (e.g., the four previous and following elements along the sequence), we implement the window definition in terms of a selected time span, enriching each sliding step with a variable number of context elements. Based on the temporal distribution of the trips, only those areas within a certain fixed time difference from the focus area are inserted into the context window. The time hyperparameter value depends on the representation purposes and the trip distribution, and its choice is closely related to the spatial resolution of the territory subdivision that is used for building the initial data sequences. Figure 1 shows a visual example of the sliding window process, depicting a context window of five minutes in the past and five in the future.

Based on the areas successively falling into the context, the model updates each focus area’s embedding vector by repeatedly performing the internal auxiliary prediction task on the space-time distribution of trips, producing a final embedding representation for each of the areas included in the “vocabulary”.

**Fig. 2:**
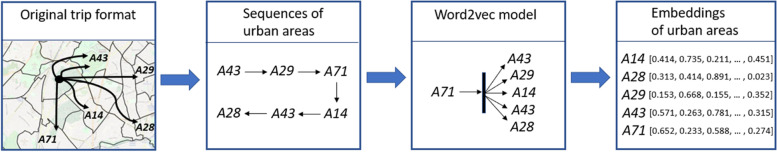
Mobility embedding generation framework

The overall workflow from raw trip data to embedding vectors of urban areas is summarized in Fig. 2.

### POI-based functional vectors

Independently from the motion embeddings, a further vector representation of urban areas is defined in parallel, with the goal of exploring a possible relation between mobility-based homogeneous regions and urban functional characteristics, attempting on providing a quantitative and qualitative evaluation of the consistency between the two aspects. These functional characteristics are modeled in the form of POI distributions, as urban POIs reflect, to some extent, the spatial allocation of urban functions and travel purposes (Bao et al., 2020; Miaoyi et al., 2018).

Given an initial set of POIs identified by distinctive categorical types and geospatial coordinates, each point of the set is associated to a specific area of the urban territory, referring to the same subdivision utilized for defining mobility embeddings. Each area, therefore, besides being described by a certain motion vector, is also connected to a characteristic POI-based representation.

In practice, the process consists of manually defining a vector of POI-category distribution for each urban area, generating a real-valued representation that allows conveying quantitative similarity measures. Following a pre-definition of a finite set of classes to associate to each POI, the POI-based vectors are constructed in the following way. Given a set of *M* POI-categories, each urban area is described by a vector of *M* dimensions, whereby each element in the vector refers to a specific corresponding unique category. The value of each element reports the number of POIs (scaled between 0 and 1), falling within that specific urban area, and belonging to the class associated to that specific vector slot.

**Fig. 3:**
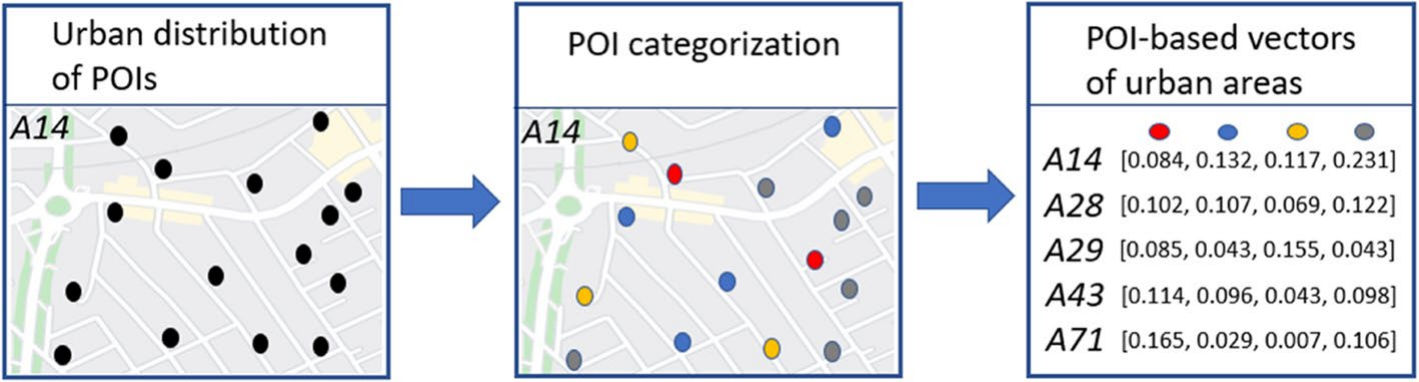
POI-based vector definition framework

This simple approach presents the advantage of explicitly defining the categories of interest, collectively counting the expression of each distinctive component and, consequently, determining characteristic vector representations that can relate with each other through distance-based statistical metrics. Figure 3 summarizes the process of POI-based vector definition.

## Experiment

This section describes the dataset, the experimental settings, and the modeling results, presenting a comparative evaluation of mobility embeddings with respect to a purely geographic perspective, and highlighting the differences between spatial and behavioral proximities.

### Data

The experimental part was carried out on a real-world dataset. Specifically, we trained the model on taxi trip recordings across New York City territories, a popular open data source already utilized in a variety of mobility-related studies (Jindal et al., 2017; Kankanamge et al., 2019; Wang & Ross, 2019; Xu et al., 2021).

New York City covers an urban area of 784 *km*^2^, administratively divided into 5 boroughs and 263 zones. Taxi trip data were acquired from the open data portal of the New York City municipality^1^, reporting the space-time individual trip information (origin-destination pairs) provided to the NYC Taxi and Limousine Commission by technology providers authorized under the Taxicab & Livery Passenger Enhancement Programs. In particular, our experiments leveraged the yellow taxi data in the whole month of March 2019.

Each trip observation is originally identified by the date and time stamp of occurrence, the departing geographic location, and the place of arrival. Both origin and destination information are indicated at the spatial resolution level of the 263 city zones. We collected a total of 7.8 million urban trips, further subjected to the pre-processing phase preceding the model training, leading to reshaping the original data format into 263 origin-based input sequences for generating 263 destination-based embedding vectors. Different choices of urban division are anyway allowed, often constrained by the used data source. An exemplifying overview of the original data features utilized in the experimental part is shown in Table 1.

**Table 1:** Exemplifying overview of the trip data format

Trip time	Origin_ID	Destination_ID
2019-03-01 00:25:27	95	130
2019-03-01 00:05:21	249	28
2019-03-01 00:48:55	138	98
2019-03-01 00:11:42	48	48
…	…	…

Regarding the POI data utilized for assessing the functional consistency of mobility-related regions, we made use of the OpenStreetMap dataset, which includes a total of 32164 unique POIs structured in 117 low-level classes, spatially distributed across the New York City territory. The pre-processed format of such data is shown in Table 2, where each POI was associated to a specific area of the territory division described above.

**Table 2:** Exemplifying overview of the POI data format

OSM_ID	POI type	POI name	Zone name	Zone_ID
357620442	restaurant	Dolcino Trattoria Toscana	Kips Bay	137
357620536	fountain	Pulitzer Fountain	Midtown North	163
357620571	school	Cathedral Preparatory Seminary	Upper West Side North	238
357620584	memorial	Carrere Memorial	Manhattan Valley	158
…	…	…		…

### Experimental settings

The model implementation was set up with an embedding size of 25 dimensions and a context window size of five minutes in the past and five minutes in the future, leveraging a mini-batch optimization training process with noise-contrastive estimation loss and Adam optimizer (Kingma & Ba, 2014; Mnih & Kavukcuoglu, 2013).

To quantitatively measure the relatedness between urban areas in terms of embedding representations and POI-type vectors, we made use of the cosine similarity metric, therefore transforming the relative associative strength into the cosine of the angle between vectors: similarity decreases as the angle grows and vice-versa. The calculation relies on the dot product between unit-normalized vectors:

$$Cos\left(\boldsymbol{a},\boldsymbol{b}\right)=\frac{\boldsymbol{a}\bullet \boldsymbol{b}}{\left\Vert \boldsymbol{a}\right\Vert\ \left\Vert \boldsymbol{b}\right\Vert\ }$$

A qualitative visualization of the embedding space was achieved through the t-distributed Stochastic Neighbor Embedding (t-SNE) algorithm (Van der Maaten & Hinton, 2008), reducing the multi-dimensional space into two dimensions while keeping similar elements close and dissimilar ones apart.

### Evaluation

We organized the research findings on two levels: a general exploration of similarity-based measures between embeddings of urban areas, and a comparison of their derived functional homogeneity identification with respect to a purely spatial proximity perspective, highlighting the captured information differences.

The first level introduces the conceptual relevance of urban areas’ mobility-based vector similarities, directly investigating the outcome of the embedding model. Examples of inter-area relatedness are included, revealing spatial-temporal links across sub-regions within the original territory division. The second level, on the other hand, focuses on a comparative evaluation of the mobility embedding method against a purely geographic approach. The underlying idea consists of exploring and comparing the functional consistency of urban region aggregations defined by mobility-based representations versus spatial distance-based definitions, evaluating advantages and disadvantages in identifying urban functional relatedness.

#### Urban areas’ mobility embeddings

The analysis output is represented by the generation of embedding vectors of single urban areas, whose mutual relations are quantified by vector similarity measures, depicting a network of spatial-temporal connections and mobility relevancies. In particular, the constructed representation relates those frequent trip destinations originating from same starting areas at similar points in time. Two areas with a high embedding similarity reflect a substantial spatial-temporal relatedness in terms of frequent co-occurrences along the same departing area’s pre-processed time series. Mobility relations between regions are therefore encoded into a common “urban vector space”. This allows grouping urban areas in a way that goes beyond the mere geography and spatial proximity. Locations that are close in space do not necessarily imply the same mobility characterization; analogously, areas that are more distant can instead potentially share similar spatial-temporal patterns.

**Fig. 4:**
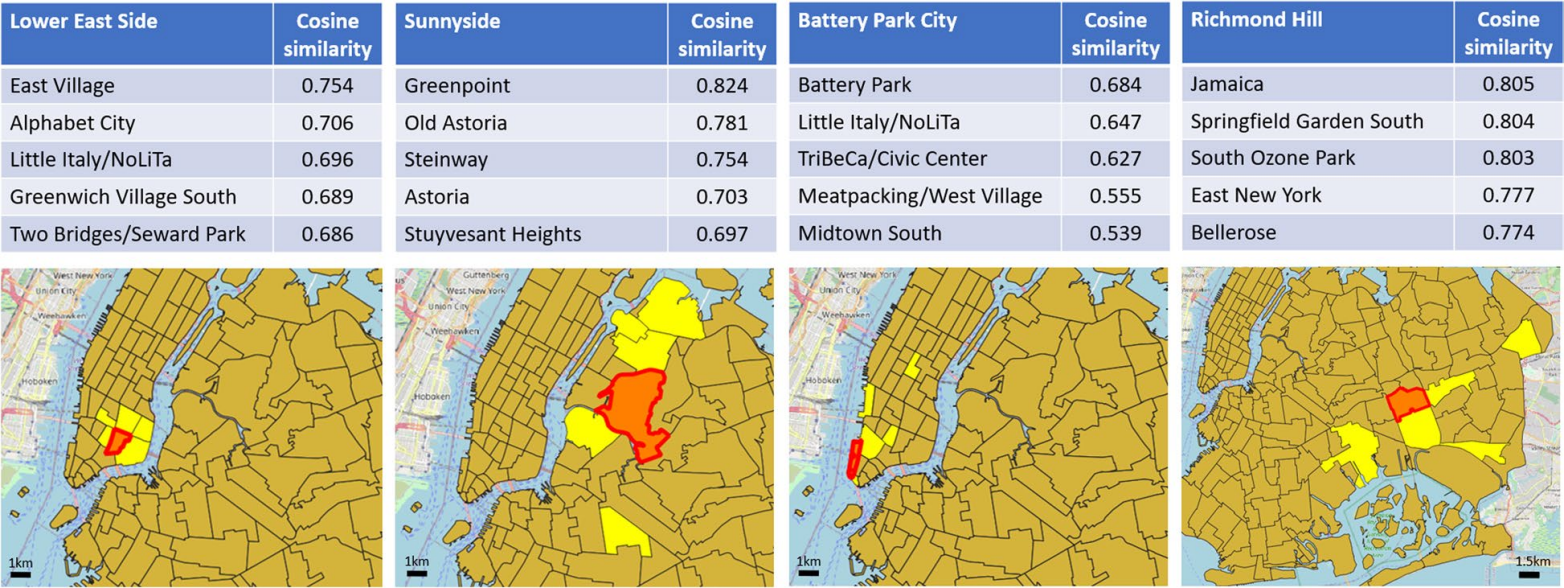
Four examples of top five similar areas (yellow regions), in terms of cosine similarity score, of a chosen reference area (red region)

Figure 4 reports four exemplifying cases listing the top five similar areas, in terms of cosine similarity score, of a chosen reference area, depicted with their geographic location on the map.

At a first glance, the mobility relatedness between urban areas seems to be influenced, to some extent, by their geographical distance. This is generally true when, intuitively, neighboring areas have similar functional characteristics, which may determine a dense web of mutual correspondences between trips’ origins and destinations. However, the connection between motion relatedness and geographic distance is not straightforward, neither in terms of presuming that all neighboring areas share the same functional characteristics, nor in terms of assuming that those characteristics necessarily affect mobility in the same way. Although a subtle trend shows up in the examples, the reported similarities do not strictly follow precise geospatial properties (e.g., Sunnyside’s top similarities do not develop on its south-east side; Richmond Hill’s ones do not develop on its north-west side). Furthermore, individual characteristics of mobility embeddings also reflect in their similarity distribution, particularly noticing the case of Battery Park City, which exhibits substantially lower top similarity scores compared to the other three cases, therefore expressing a general weaker tendency of sharing similar mobility patterns with the other regions.

**Fig. 5:**
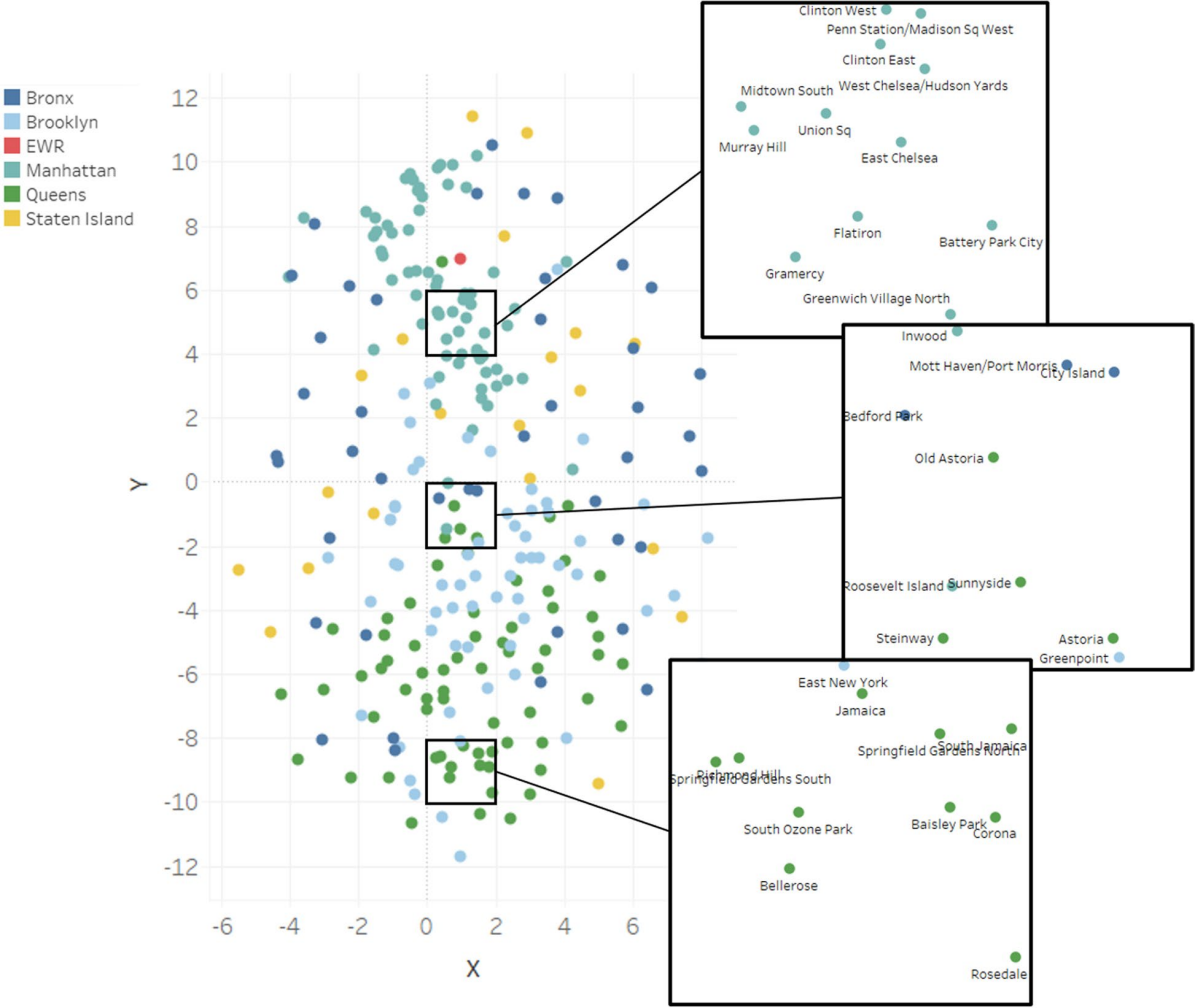
Two-dimensional t-SNE reduction of the urban areas’ vector space

To visually display an overall representation of the vector space and the motion relations between the totality of urban areas, embeddings can be reduced through t-SNE into two dimensions, as displayed in Fig. 5. Each area’s label in the plot is differently colored according to the borough it belongs to. All in all, the tendency of grouping neighboring regions is present, but many exceptions are distinctly observable. For a better comprehension, three portions of the vector space are zoomed in, displaying a mixture of urban areas.

To sum up, we highlight the fact that different non-adjacent areas may anyway be strongly related from a mobility-based origin-destination perspective, consequently locating in the same part of the embedding space. Going beyond spatial proximity, the vector space reveals a complex system of inter-area relations, revealing hidden spatial-temporal patterns through a convenient dynamic data-driven representation of urban areas. Frequent co-occurrences of destination areas, over time, originating from the same departing area, are therefore translated into mobility-based associations and, consequently, into an index of situational relatedness within a potential context of trip-related perception.

#### Functional consistency within mobility-related urban areas

A quantitative evaluation for assessing the functional consistency of mobility-based representations of urban areas was carried out by determining, for each given area, a characteristic urban cluster (in the form of a combination of multiple areas) based on the top similar embedding vectors. The task aims to verify whether the POI distribution within that identified urban cluster is consistent with the one of its given target area. Specifically, the idea is to compare the average intra-cluster similarity with the extra-cluster one, expecting higher values in the first case and, consequently, relating the functional-based POI distribution with the motion behavior of people.

From a practical perspective, the workflow consists of specifying a POI vector, based on a pre-defined choice of functionality-related aspects, and calculate its cosine similarity with respect to the other urban regions’ POI vectors, grouped according to the previously calculated mobility embeddings’ similarities. For instance, if the mobility embedding of area *Q* identifies a cluster of top similar areas including *R*, *S*, *T*, and *W*, the functional evaluation is based on measuring the POI vector similarity of area *Q* with any other urban area, and compare the average similarity with *R*, *S*, *T*, *W* against the average with all other areas. If the first measure is higher, the assumption is that mobility embeddings intrinsically model urban functionality additionally to their motion-related representation.

To verify the results, we compared these outcomes with a purely geographic-based approach, namely defining aggregated urban clusters only based on the spatial distance between areas, in contrast to our mobility-based top similar embedding representation approach. The dual similarity comparison (intra-cluster vs extra-cluster), in this case, was therefore set up simply as closer regions vs distant regions. The underlying idea relied on assessing if mobility-based territory aggregations may provide more information on the urban functionality rather than a purely geospatial proximity perspective.

**Fig. 6:**
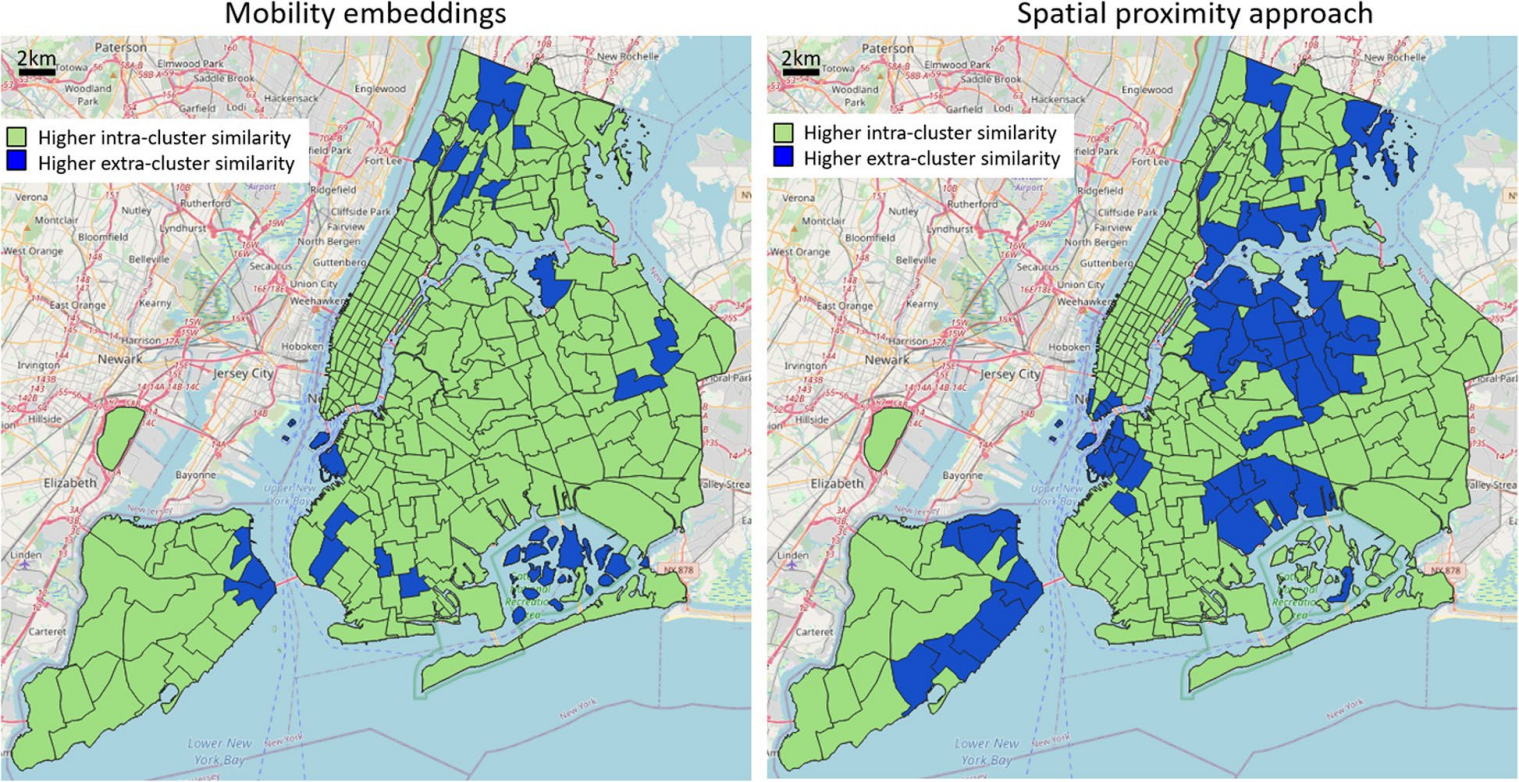
Assessment of functionality cluster consistency according to residential and tourist-related functionality classes. Results are reported in terms of “winning” or “losing” similarity scores for each reference area (green if its intra-cluster similarity is higher than its extra-cluster one; blue if vice-versa)

Inspired by previous research efforts (Yuan et al., 2021; Zhang et al., 2018), we explored different combinations of POI-type categorizations, to deliver various insights on the experimental trials. We first proceeded to categorize each POI type into one of two possible classes: residential (e.g., schools, laundries, hairdressers, beauty shops, jewellers, convenience stores, furniture shops, supermarkets, bakeries, pharmacies, doctors, dentists, opticians, ...) and tourist-related functions (e.g., attractions, memorials, tourist info centers, hotels, toilets, ...). Figure 6 illustrates the results in terms of “winning” or “losing” similarity scores for each reference area (green: intra-cluster similarity is higher than extra-cluster one; blue: vice-versa). The similarity threshold value to define the mobility-based clusters and the distance threshold value to define the geographic-based clusters were both set to maximize the intra-cluster similarity. The maps reveal that 242 out of 263 areas have a higher intra-cluster similarity in the case of mobility embeddings, while only 198 in the geographic-based case. This suggests a strong tendency of embeddings of implicitly grasping information on the resident/tourist duality, which is better inherently described by trip-based vectors rather than the simple geographic distance. Moreover, it is clear how the errors of the geographic approach tend to aggregate spatially, symptoms of the presence of neighboring areas in space having different POI distributions. The mobility embedding approach, on the other hand, is not directly influenced by spatial proximity, modeling functional regions only based on homogeneous people’s movements.

**Fig. 7:**
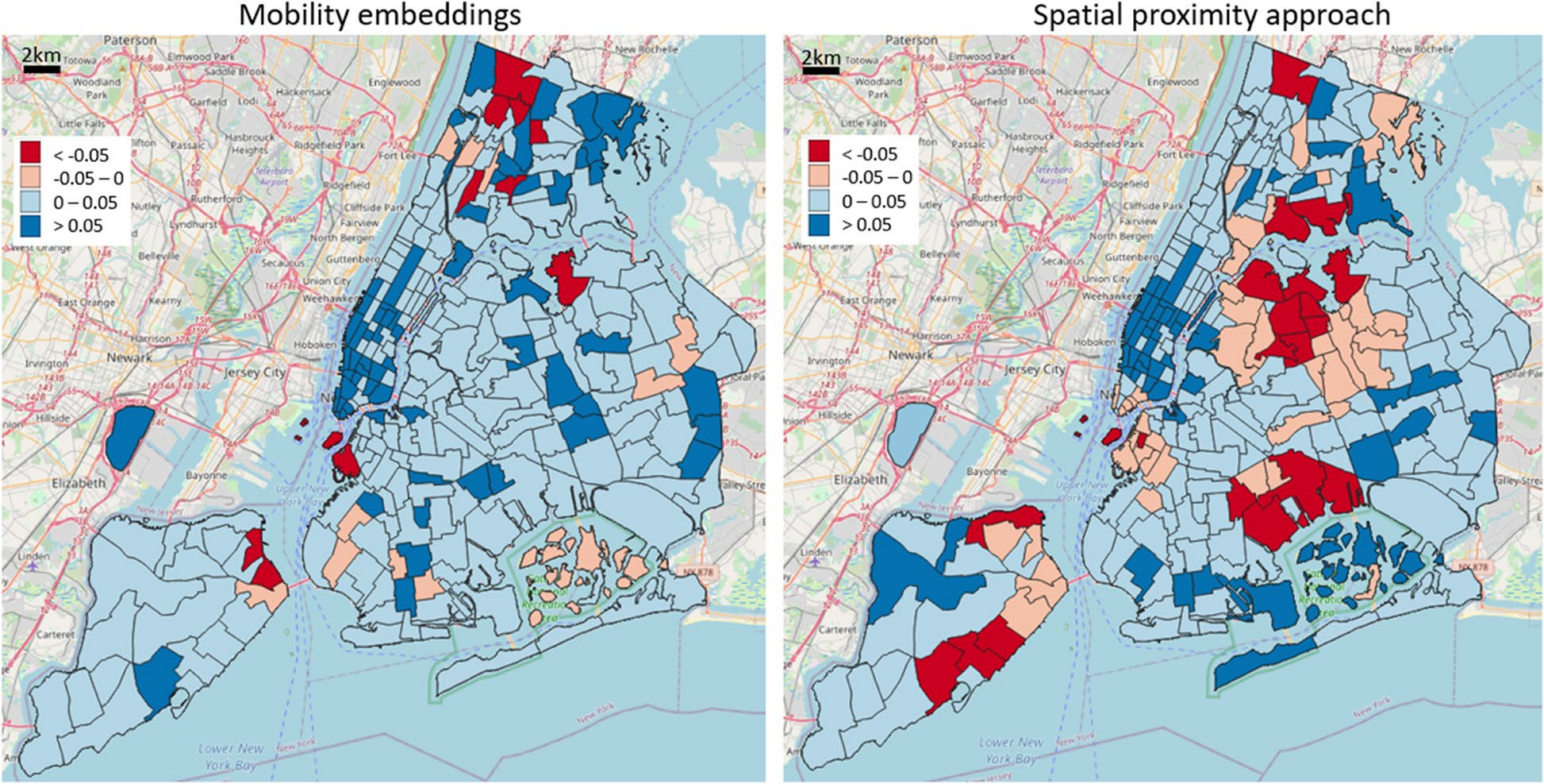
Assessment of functionality cluster consistency according to residential and tourist-related functionality classes: cosine similarity difference between intra- and extra-cluster averages for each reference area

A deeper investigation reveals the quantified cosine similarity difference between intra- and extra-cluster averages for each reference area, as shown in Fig. 7. In particular, it is relevant to notice that Manhattan area exhibits higher values in both the mobility-based and geographic case, consequence of a spatial proximity influence on the POI distribution over the territory. The geographic approach instead presents substantial errors in the Queens borough and its neighboring areas, whereby the mobility embeddings are not particularly affected, delivering a portray of diverse functional characteristics among geographically proximate regions.

**Fig. 8:**
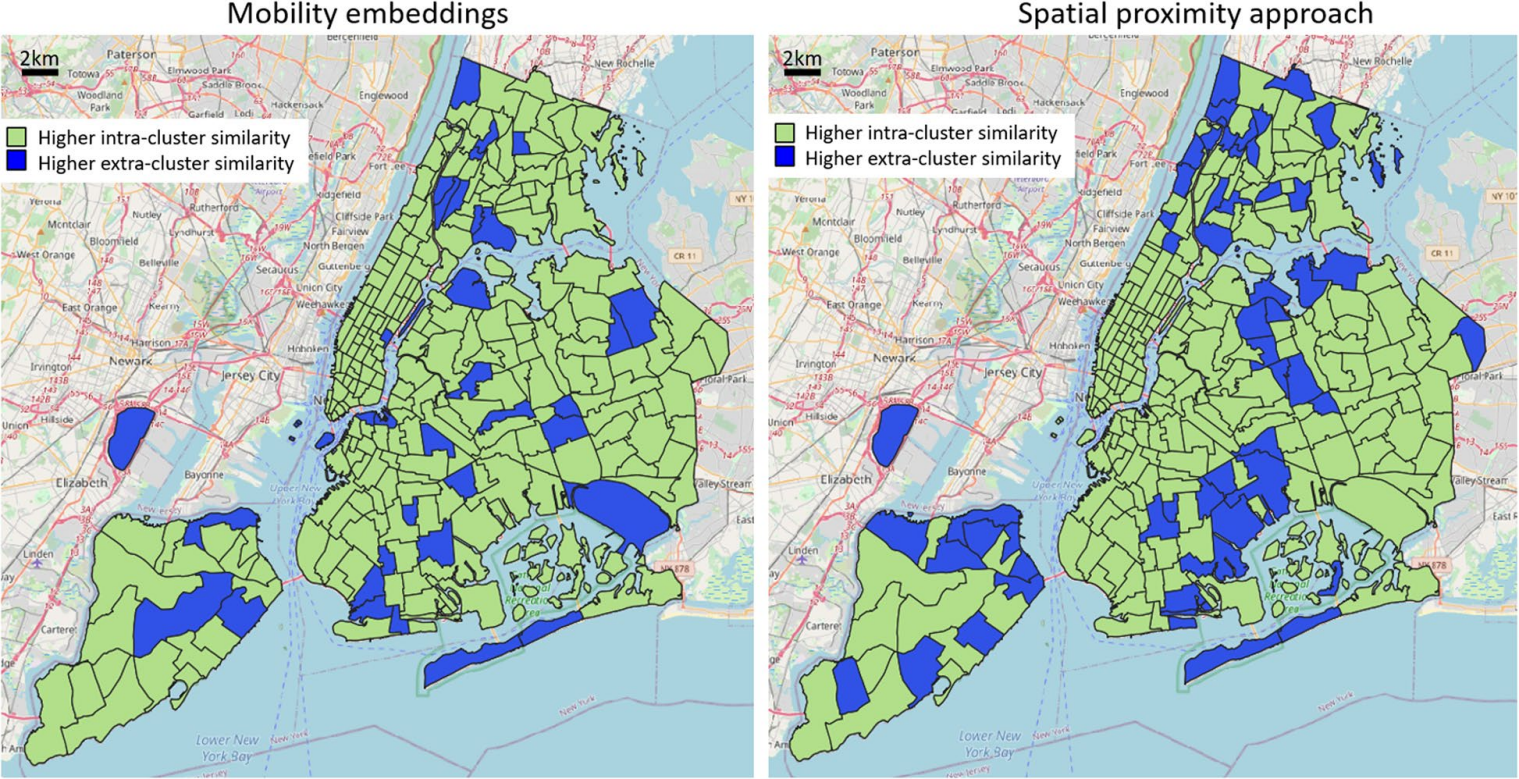
Assessment of functionality cluster consistency according to different dining-related POI classes (fast food, restaurant, pub, café). Results are reported in terms of “winning” or “losing” similarity scores for each reference area

Further POI vector definitions may also be investigated. Instead of a general categorization into resident and tourist-related functionalities, more detailed classifications can be explored, delivering different outcomes and derived assumptions. An option could be to study a specific combination of POI types, for instance related to dining activities. Specifically, we proceeded to select a subset of four separate classes of POIs: fast food, restaurant, pub, café. The goal was to investigate mobility-based clusters with regard to the distribution of such four dining options. Figure 8 reports the winning (intra- or extra-cluster) similarity score for each reference area, revealing that 227 areas have a higher intra-cluster similarity in the case of mobility embeddings, while only 209 in the geographic-based case. Again, we can observe that the errors of the geographic approach tend to aggregate spatially, reflecting different POI-type distributions across neighboring areas. Such spatial aggregation is not present in the mobility embedding case, which models functional regions based on human movements rather than geographic proximity.

**Fig. 9:**
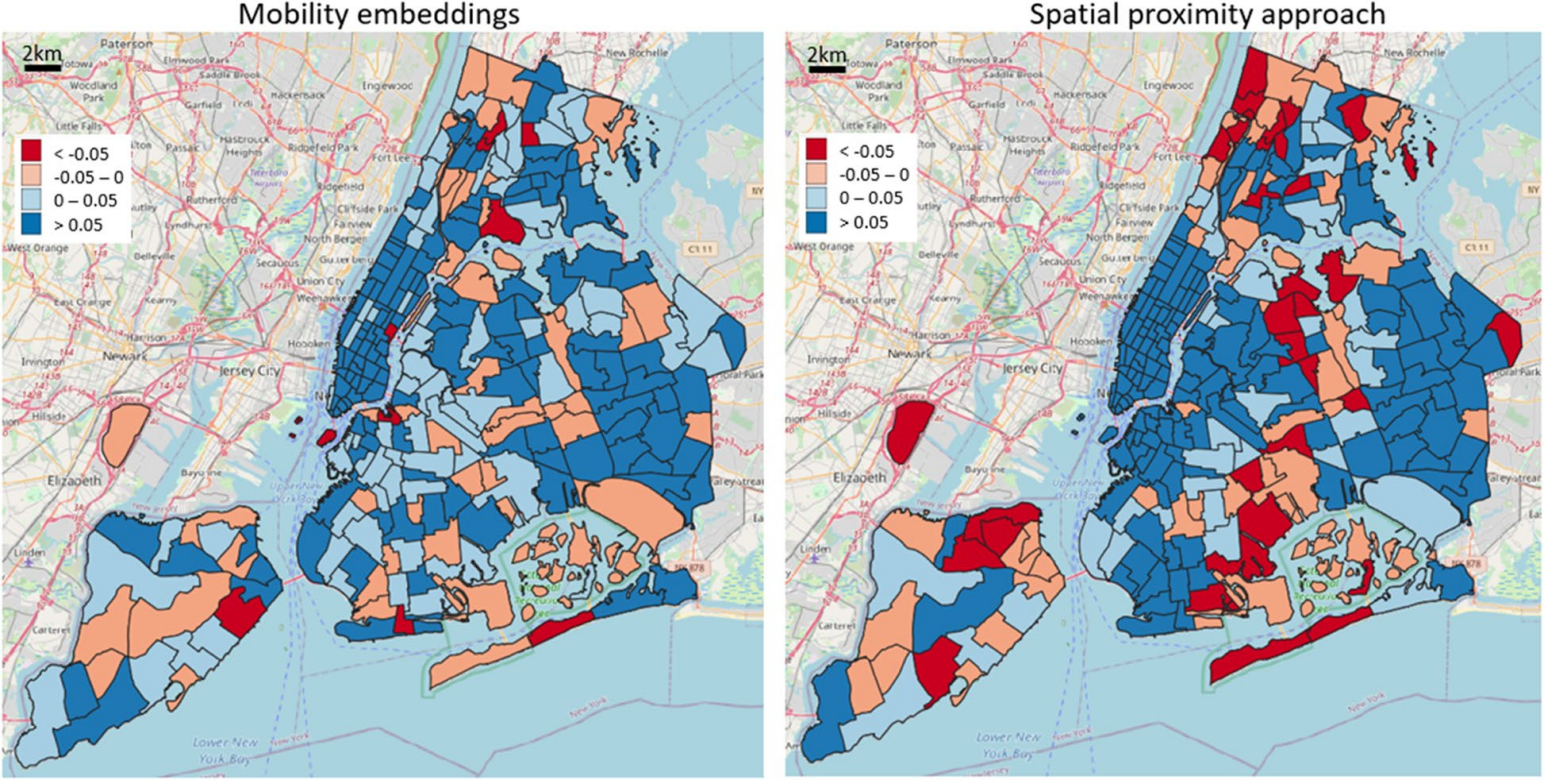
Assessment of functionality cluster consistency according to different dining-related POI classes (fast food, restaurant, pub, café): cosine similarity difference between intra- and extra-cluster averages for each reference area

By plotting the cosine similarity difference between intra- and extra-cluster averages, as shown in Fig. 9, we observe that the geographic case exhibits widespread extremes, namely a larger number of high positive values but also a substantial number of very negative values, whereas the embedding approach is more balanced. This outcome depicts how mobility and geography gives different responses to the complex phenomenon under study, delivering an intricate portray of diverse dining characteristics among urban regions.

**Fig. 10:**
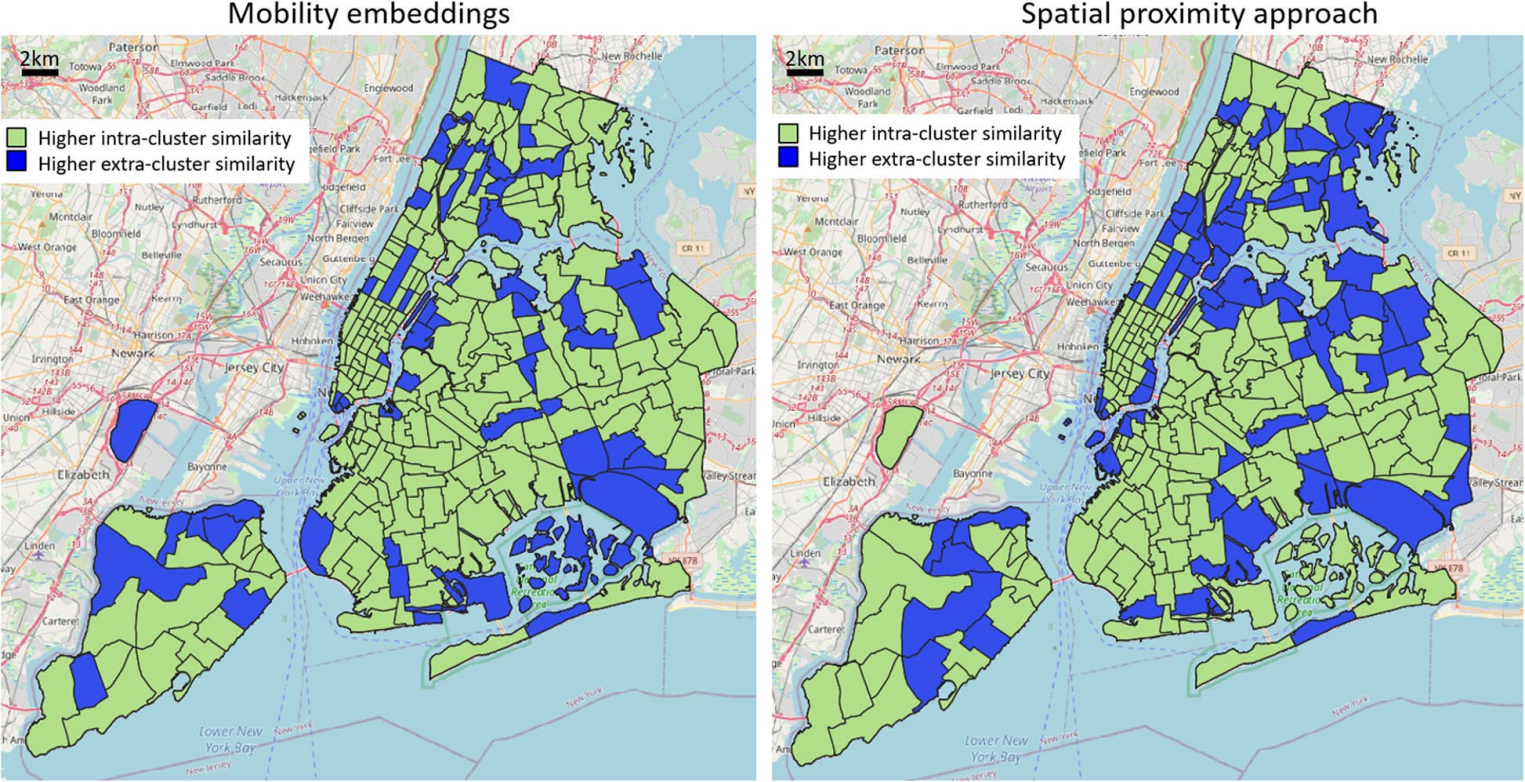
Assessment of functionality cluster consistency according to different POI-based functionality classes (cater, public facility, shopping, life service, accommodation service). Results are reported in terms of “winning” or “losing” similarity scores for each reference area

Finally, to provide a further example on the variety of potential combinations and explorations of POI categories (each revealing different insights and patterns), we report a last trial classifying POIs into one of five possible classes: cater (e.g., fast foods, restaurants, bars, cafés, pubs), public facility (e.g., benches, water supplies, toilets, waste baskets), shopping (e.g., convenience stores, clothing shops, supermarkets, shoe shops, furniture shops), life service (e.g., hairdressers, laundries), and accommodation service (e.g., hotels). By investigating mobility-based clusters with regard to the distribution of those five classes, the winning scores, reported in Fig. 10, state that 204 areas have a higher intra-cluster similarity, against a geographic-based result of 189 areas. Once again, spatial aggregations are more prominent on the geographic side, even if the performance diversity is not as large as in the previous two examples.

**Fig. 11:**
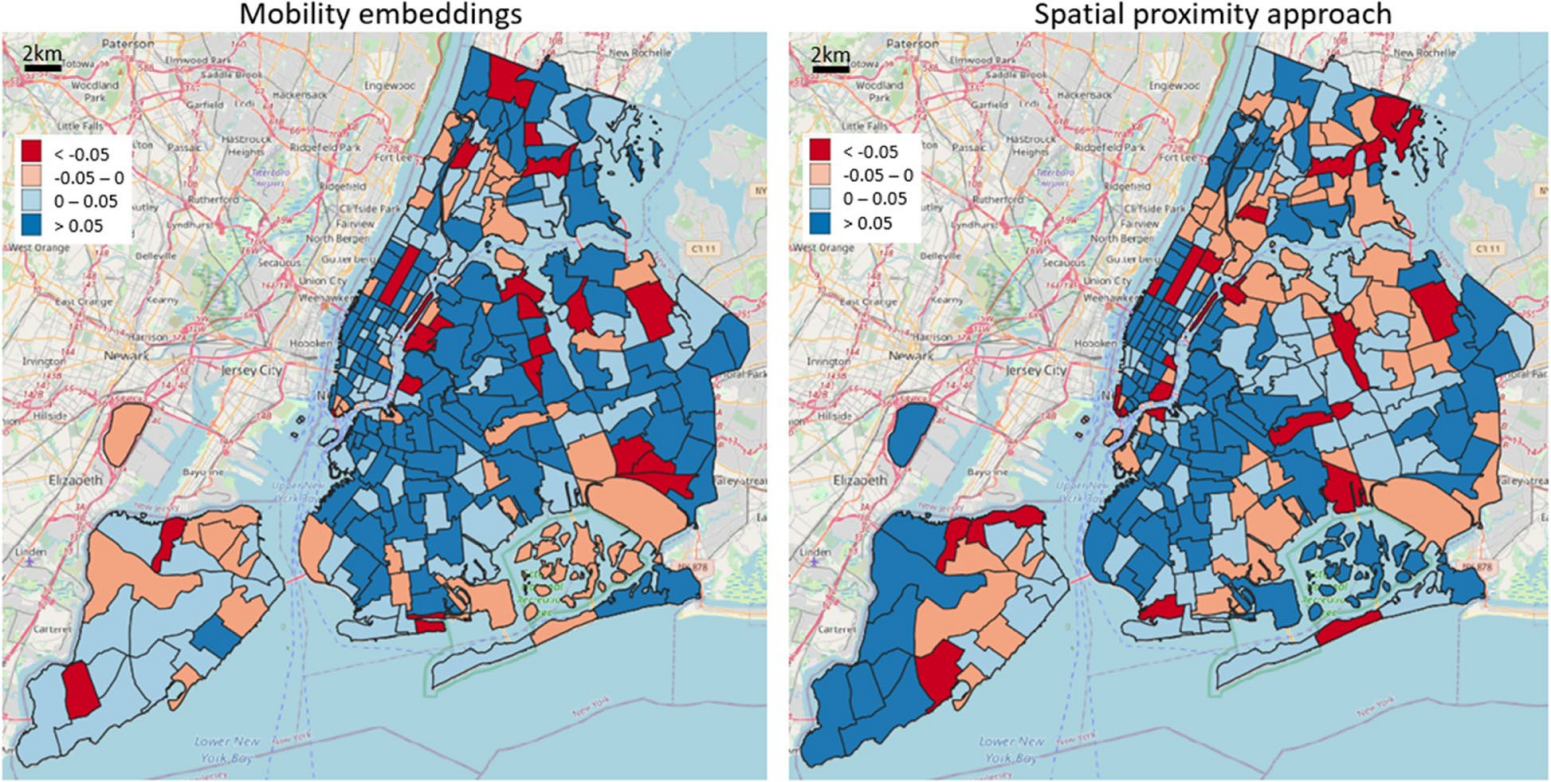
Assessment of functionality cluster consistency according to different POI-based functionality classes (cater, public facility, shopping, life service, accommodation service): cosine similarity difference between intra- and extra-block averages for each reference area

The cosine similarity difference between intra- and extra-cluster averages, reported in Fig. 11, reveals a tendency of the geographic approach of obtaining slightly higher positive values over Manhattan, consequence of a similar class distribution among proximal areas in space; however, the rest of the city follows a general opposite trend, with the embedding approach disclosing higher positive values, therefore implying that similar and diverse functional characteristics are better grasped based on the movement of people rather than the mere geographical proximity.

## Discussion and conclusion

Within the modern perspective of cities as integrated systems of multiple interacting networks, human mobility represents a central factor for gaining insights on effective urban structures and functionalities. Going beyond the simple geography, the spatial-temporal traits of people’s movements are intended to carry an implicit message on the functional relationships between different urban areas, which not necessarily follow neighboring spatial properties. Aiming to grasp this meaningful aspect of the urban configuration, we proposed a data-driven experimental approach for investigating possible correlations between travel patterns and urban functions.

The research framework was characterized by similarity measures between multi-dimensional vectors of urban areas, constructed via unsupervised machine learning on the sole basis of trips’ occurrences in space and time. Specifically, we organized trip-related destination areas into spatial-temporal sequences, subsequently fed into a time-dependent Word2vec-based model to accordingly generate embedding representations. This outcome was then compared with the POI-type distribution across the city, to assess the functional consistency of mobility clusters of urban areas.

By embodying the concept of spatial-temporal relatedness into a mathematical representation, whereby related areas end up gathering next to each other in a multi-dimensional vector space, mobility embeddings disclosed a complex system of relations, depicting a network of space-time connections and relevancies. Whereas motion relatedness between urban areas can be influenced, to some extent, by their geographic distance, we revealed that the link “mobility-proximity” is not straightforward, giving rise to a dense web of mutual correspondences and different similarity distributions expressing weaker or stronger tendencies of sharing similar mobility patterns with other regions.

Moreover, an evaluation of functional consistency was carried out by determining, for each urban area, a characteristic “mobility block” identified by its top similar motion embedding vectors, and further comparing the average POI-type intra-block distribution similarity with the extra-block one. We analyzed three examples of POI categorization (tourist/resident duality, dining activities, high-level functional types), and set up a comparative baseline as a purely geographic-based approach, namely defining aggregated urban clusters only based on spatial distances. The results emphasized how the errors of the geographic model tended to cluster spatially, symptoms of the presence of neighboring areas in space having different POI distributions; on the other hand, this rarely happened in the mobility embedding case, not directly influenced by spatial proximity but built on people’s homogeneous movements. General observed characteristics comprise: a higher quantity of intra-cluster similarity samples in the mobility-based approach, a substantial number of very negative values of intra-extra cluster difference in the geographic case, different overall responses of the two models towards the intricate portray of diverse POI characteristics among urban regions. According to the use case under study, whereas Manhattan area expresses similar internal functional characteristics, and therefore the geographic approach was very effective, the rest of the city depicts a complex diverse pattern of POI-type distribution, making the embedding solution substantially better.

To sum up, the main contribution of this study lies in providing an effective approach for exploring the relations between mobility and functionality through machine-readable representations able to convey similarity measures. By automatically mining the underlying relations of trip data in terms of characteristic destinations from same urban regions at similar points in time, we provide a novel perspective of approaching human mobility and urban activity, exploring the relationship between people’s motion and the mixture of basic functional units across the urban space. Applicable to any arbitrary spatial-temporal scale and in presence of any initial territory division, the methodology allows for direct comparisons and POI-based associations of mobility-related areas, providing a tool for an alternative investigation of homogenous urban regions and paving the way for ideally identifying urban functional zones only based on the movement of people.

The potential extensions of this paper may comprise multiple perspectives. A major direction can deal with expanding the inherent limitation of the tested case study. Whereas we tackled human mobility processing on the perspective of taxi trip data, the methodology could be applied, in principle, to any kind of trip-related format. We assumed taxi travels to be an acceptable approximation of motion behavior for studying urban functional characteristics, though depicting only one of the multiple complex facets linking human mobility and urban morphology. Different data sources may be employed to further investigate how various transportation modalities are able to catch different aspects of the way a certain urban area is “utilized”. Such combination may be explored in the form of a collective data merging or even as a comparative analysis on how several travel demands can differently relate to the POI-type distributions over the territory.

In addition, mobility embeddings can be tested in various applications, either as a basis for clustering and similarity searching, or even as an input to downstream predictive models, potentially merged with further data sources into more complex representations. Moreover, the experimental part may leverage various implementation options, such as different resolutions, in time and space, and study areas of variable size, attempting on modeling different scales of the hidden spatial-temporal aspects of the urban reality. A variety of POI-type categorizations can further be explored, investigating and analyzing the amount of information that mobility embeddings provide with respect to diverse functional configurations. Another matter that may be worth taking into account is the study of the seasonality and trend evolution over time, whereby we dealt with a static use case. Finally, whereas our data-driven model implicitly catches an ensemble outcome of the complex urban aspects of the intrinsic relation between human mobility and urban functionality, the analysis and reasoning around single deeper issues and contributions require a prominent theory-driven exploration, aiming to merge theoretical-based assumptions with data-based evidence.

To conclude, the proposed perspective is intended to complement traditional studies on the modeling of urban activities, presenting a landmark for the automatic mining of mobility and POI data types within the context of urban management and planning.

## Data Availability

The taxi trip data used in the current research work can be acquired from the open data portal of the New York City municipality (https://www1.nyc.gov/site/tlc/about/tlc-trip-record-data.page).

## Footnotes

1 https://www1.nyc.gov/site/tlc/about/tlc-trip-record-data.page
